# Acute limb ischemia secondary to left brachial artery occlusion in a 45-year-old woman: A case report

**DOI:** 10.1097/MD.0000000000046515

**Published:** 2025-12-12

**Authors:** Osman Zada, Tirath Patel, Suliman Syed, Muhammad Suhaib Hanif, Ayesha Haq, Nikhilesh Anand

**Affiliations:** a Saidu Medical College, Swat, Pakistan; b Department of Surgery, Trinity School of Medicine, Kingstown, Saint Vincent and the Grenadines; c Peshawar Medical College, Peshawar, Pakistan; d Dow University of Health Sciences, Karachi, Pakistan; e Aga Khan University Hospital, MBBS-BUHSCK, Karachi, Pakistan; f Department of Medical Education, University of Texas Rio Grande Valley, Edinburg, TX.

**Keywords:** acute limb ischemia, brachial artery occlusion, case report, chronic hypertension, embolectomy

## Abstract

**Rationale:**

Acute limb ischemia is a vascular emergency that occurs when blood flow to a limb suddenly decreases, putting the limb at risk if not treated promptly. Acute limb ischemia accounts for 11% of all vascular emergencies, among which arm ischemia is relatively rare. It is caused by occlusion of the brachial artery, most commonly by vascular thromboembolism. We report a female patient with left upper limb ischemia induced by occlusion of the brachial artery.

**Patient concerns:**

We report a 45-year-old female who presents with an acute onset of severe pain, numbness, and pallor in her left arm. Pulse was absent, and muscle power was decreased on physical examination. The patient’s past medical history was significant for chronic hypertension controlled with medication.

**Diagnoses:**

The diagnosis of acute left arm ischemia was confirmed based on the history of hypertension and Doppler ultrasound, which showed occlusion of the left brachial artery due to atherosclerotic changes.

**Interventions:**

The patient was initially treated with heparin and analgesics for pain control. Embolectomy was performed under local anesthesia, and a proximal longitudinal incision was made. The brachial artery was exposed, the proximal artery was clipped, the radial and ulnar arteries were cleared using a Fogarty balloon catheter, and the arteriotomy was closed with Prolene 6/0. Hemostasis was secured, and the skin was closed with Prolene 3/0. The patient was given Augmentin 1-g tablet “Quaque Die” means once daily (QD), heparin injection QD, Tonoflex P injection QD, and Loprin 75-mg tablet QD.

**Outcomes:**

During the 8-week follow-up period, all patient symptoms improved. The peripheral pulse was present; there were no paresthesia or numbness, except for mild pain in her left thumb due to ischemia. This was a postoperative complication due to the lodging of an embolic fragment from surgery.

**Lessons:**

Early diagnosis and prompt surgical intervention in brachial artery occlusion are essential to prevent complications such as gangrene, even in patients without overt vascular risk factors.

## 1. Introduction

Peripheral artery disease (PAD) is a progressive atherosclerotic vascular disease characterized by stenosis and occlusion of arteries, associated with high rates of cardiovascular events and death, affecting around 200 million individuals globally.^[1]^ Acute limb ischemia (ALI), a major vascular emergency, is defined as a sudden decrease in limb perfusion that threatens limb viability.^[2]^ ALI is a medical/surgical emergency that ultimately risks the patient’s life as well as the limb.^[3]^ Early diagnosis and rapid restoration of arterial perfusion are crucial for preserving the viability of the affected limb and preventing irreversible tissue damage, which can result in life-threatening complications.

The clinical presentation may be highly variable, and typical symptoms may be absent in a significant number of patients.^[4]^ Symptom onset may occur within minutes or progress over hours to days. The spectrum ranges from intermittent claudication to severe rest pain, paresthesia, muscle weakness, paralysis, and, in advanced cases, gangrene. Most patients initially present with intense limb pain and sensory changes. As ischemia persists, motor deficits such as weakness or paralysis, along with sensory loss and changes in skin color, become more pronounced due to progressive tissue compromise.^[5]^ Despite recent advances in medical and surgical practices, studies show that acute limb ischemia continues to have a 30-day amputation rate of 10% to 30%, a mortality rate ranging between 9% and 25%, and an inferior 1-year amputation-free survival of only about 50%.^[6]^ Acute upper limb ischemia is a relatively uncommon clinical manifestation of thromboembolism, most commonly caused by embolic events, particularly from cardiac sources such as atrial fibrillation, valvular disease, or mural thrombi.^[7]^ There is a significant lack of comprehensive data regarding ALI of the upper extremity, particularly when caused by brachial artery occlusion, likely due to its relative rarity compared with ALI of the lower limb. Nonetheless, upper limb ALI carries a substantial risk of morbidity and can cause severe functional impairment, tissue necrosis, and even limb loss if not promptly managed.^[8]^

This case report seeks to highlight the clinical significance of upper limb ALI due to brachial artery occlusion. It aims to draw attention to its underrecognized presentation and variable etiologies, emphasizes the need for the same level of diagnostic vigilance and therapeutic urgency that is applied to lower limb ALI, and encourages further study into the standard and optimal management of upper extremity brachial arterial occlusions.

## 2. Case presentation

### 2.1. Patient information and chief complaint

We report a 45-year-old female in our hospital’s emergency department with a chief complaint of severe pain in her left arm. According to the patient’s husband, she had a sudden onset of pain in her left arm while doing routine activities 3 hours prior.

### 2.2. Past medical and personal history

Past medical history was significant for chronic hypertension controlled with medication. She denied having any history of abortion, claudication, cardiovascular, or autoimmune illnesses. She was right-handed with no history of oral contraceptive pills use or clotting history. She had no history of trauma or PAD.

### 2.3. Physical examination

Physical examination showed temperature 37.8℃, pulse 70, BP 142/85, and respiratory rate 15 breaths/min. Examination of the hand showed absent radial and ulnar pulses in the left hand, and a regular rhythm of 70 beats per minute in the right hand. The affected arm showed signs of coldness, paleness, and numbness. Paresthesia was clinically detectable, and the muscle power grading was 1/5.

### 2.4. Investigations and diagnosis

**Figure 1.**
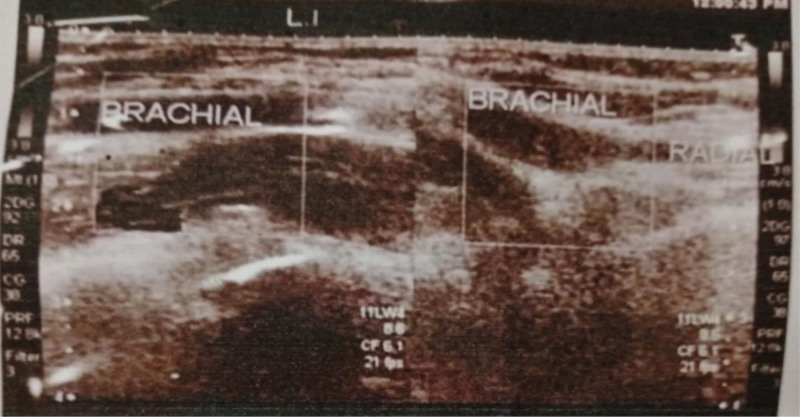
Doppler ultrasound of the left hand brachial artery indicated significant atherosclerotic changes, along with echogenic material seen in the left distal brachial artery extending into its branches (radial and ulnar arteries), completely occluding blood flow.

Laboratory data for renal function tests, liver function tests, and a hypercoagulability workup showed normal ranges. The echocardiography and complete blood count results of the patient are shown in Tables 1 and 2. The Doppler ultrasound report of the patient revealed significant atherosclerotic changes, with echogenic material in the left distal brachial artery extending into its branches (radial and ulnar arteries) and completely occluding blood flow, as shown in Figure 1. The diagnosis of acute limb ischemia was confirmed.

**Table 1:** Complete blood count.

Test	Result	Normal range
Hemoglobin	9.2 g/dL	11–16 g/dL
Total RBC count	3.83 million/mm^3^	4.5–5.5
Total WBC count	16,000/mm^3^	4000–11,000
Neutrophils	70%	40%–75%
Lymphocytes	26%	20%–45%
Eosinophil’s	2%	2%–6%
Monocytes	2%	6%–10%
Platelets	257,000/mm^3^	150,000–400,000
Mean corpuscular volume (MCV)	70 fl	83–101
Mean corpuscular hemoglobin (MCH)	24.0 pg	27–32 pg
Mean corpuscular hemoglobin concentration (MCHC)	33.9 g/dL	31–35 g/dL

RBC = red blood cells, WBC = white blood cells.

**Table 2:** Echocardiography report.

Parameters	Measured	Range
End diastolic volume (EDV)	4.4 mL	3.5–5.7 mL
End systolic volume (ESV)	3.0 mL	--------
Right ventricle (RV)	2.0 cm	0.9–2.6 mL
Interventricular septum (IVS)	1.0 cm	0.6–1.1 cm
Aorta	2.8 cm	2.0–3.7 cm
Left atrium (LA)	3.5 cm	1.9–4.0 cm
Fraction shortening (FS)	32%	28%–43%

### 2.5. Therapeutic intervention

The initial treatment consisted of a 5000 unit stat bolus IV infusion of unfractionated Heparin and a Toradol injection for pain management. Following radiographic confirmation of ALI, an urgent surgical intervention was required. Embolectomy was performed, and the operative findings were a thrombus at the level of the bifurcation of the radial and ulnar arteries. Under local anesthesia, a proximal longitudinal incision was made. The brachial artery was exposed, the proximal artery was clipped, the radial and ulnar arteries were cleared, and the Arteriotomy was closed with Prolene 6/0. Hemostasis was secured, and the skin was closed with Prolene 3/0. During her hospital stay, the patient was treated with Augmentin 1-g tablets once daily, heparin injections once daily, Tonoflex P injections once daily, and Loprin 75-mg tablets once daily. On the second postoperative day, the left brachial, radial, and ulnar artery pulses were palpable, with no signs of cyanosis. The sensory and motor assessments demonstrated complete restoration. Upon discharge, the patient was prescribed Xceft 10 mg for 2 weeks, Loprin 75 mg, and Augmentin 1 g per day for 1 month.

### 2.6. Follow-up and outcomes

**Figure 2.**
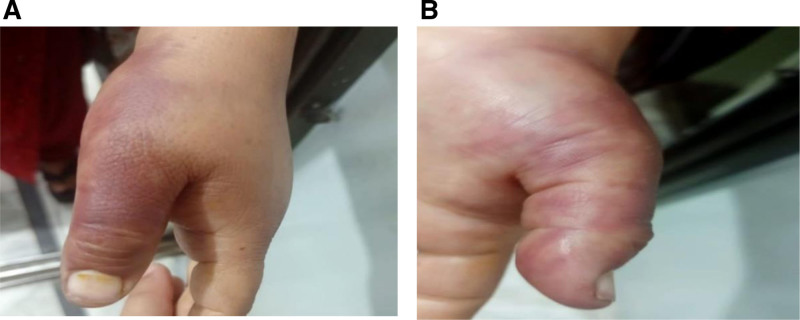
(A) Lateral view of the left thumb showing red discoloration on 10 days of follow-up. (B) Front view of the thumb showing red discoloration on 10 days of follow-up.

After 10 days, the patient’s symptoms had improved. The peripheral pulse was present; there were no paresthesia or numbness, except for mild pain in her left thumb due to ischemia. This was a postoperative complication due to lodging of an embolic fragment from surgery, and 10-day follow-up images are shown in Figure 2A and B.

**Figure 3.**
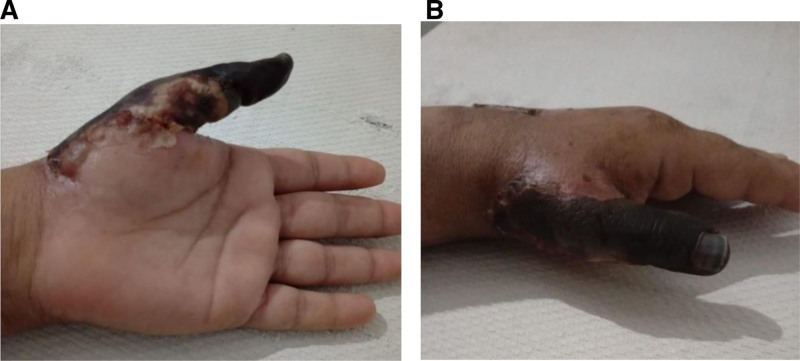
(A) Front view of the hand at 8-week follow-up showed black discoloration of the thumb, which indicates gangrene. (B) Lateral view of the left hand at 8-week follow-up showed black discoloration of the thumb, which indicates gangrene.

At the 8-week follow-up, there were no complications in the arm or on any of the fingers of the left hand, except for the distal end of the thumb, which appeared dark, almost black, indicating gangrene, as shown in the 8-week follow-up images in Figure 3A and B. Dry Gangrene is seen, which is due to tissue desiccation, blackened, demarcated necrosis (no infection).

## 3. Discussion

ALI is a vascular emergency characterized by a sudden decrease in limb perfusion, which can potentially threaten limb viability if not promptly treated. Although the lower extremities are more commonly affected, upper extremity ALI constitutes ~2% to 18% of all cases, and the distal brachial artery is a common site for embolic obstruction because it is a point of arterial bifurcation, where emboli often lodge due to a caliber mismatch between the embolus and the smaller-diameter radial and ulnar arteries.^[9]^ In our case, a 45-year-old hypertensive woman presented with sudden-onset upper limb pain, absent peripheral pulses, pallor, paresthesia, and weakness, classic manifestations consistent with the 6 P’s of ALI: pain, pallor, pulselessness, paresthesia, paralysis, and poikilothermia.^[10]^ The absence of trauma or autoimmune background, combined with normal coagulation and hypercoagulability profiles, suggests that the embolic occlusion likely resulted from atherosclerotic plaque rupture or a cardioembolic source, despite normal echocardiographic findings.

Doppler ultrasonography remains a reliable first-line modality for diagnosing ALI in the upper limb, capable of identifying both thrombotic and embolic events.^[11]^ In this case, it revealed echogenic material completely occluding the distal brachial artery and its branches. The decision to proceed with urgent embolectomy aligns with current guidelines that recommend revascularization within 6 to 8 hours of symptom onset to prevent irreversible muscle and nerve damage.^[12]^ Postsurgical outcomes were favorable, with restoration of pulses and sensory-motor function. However, the patient experienced dry gangrene as a result of ischemic injury to the thumb. This is a known risk and happened even after an embolectomy went well. Distal embolization of a tiny thrombotic fragment is the most likely cause. Even with a technically sound procedure, this can occur; however, a technical error, such as insufficient flushing of the distal arterial tree before removing the clamps and closing the arteriotomy, can exacerbate the issue. When blood flow is restored, microscopic debris that has been missed by inadequate flushing may travel to the end arteries of the digits.^[13]^ To reduce the risk of distal embolization as a surgical complication, complete thrombus clearance with multiple passes of the Fogarty balloon catheter in both the radial and ulnar arteries, with special attention to distal branches, and intraoperative angiography is recommended.

Notably, this case underscores the diagnostic challenge posed by atypical ALI presentations in middle-aged women without overt cardiac or vascular disease. The patient’s mild anemia and leukocytosis likely reflect a secondary inflammatory response rather than a primary cause. The strength of this case lies in the prompt diagnosis and timely surgical management, which allowed limb salvage despite delayed presentation. However, a key limitation was the lack of advanced vascular imaging, such as CT angiography, which might have better delineated the clot burden. Furthermore, the development of thumb necrosis suggests a need for better distal assessment during and after embolectomy. Early diagnosis, appropriate anticoagulation with unfractionated heparin, and surgical intervention were essential to the positive outcome. Postoperative antibiotic coverage helped prevent secondary infections, especially in ischemic tissue areas.^[14]^

## 4. Conclusion

This case highlights the importance of early clinical recognition of ALI, even in patients without classic risk factors such as PAD, coagulation disorder, and oral contraceptive pills use. Prompt intervention can lead to favorable outcomes and decrease the risk of mortality. Clinicians must maintain a high index of suspicion and be prepared to act quickly. Early embolectomy was lifesaving, restoring perfusion, and preventing significant tissue loss. Additionally, careful postoperative monitoring is vital to identify complications such as distal embolization or gangrene, as evidenced in this patient’s delayed thumb necrosis. This case highlights the importance of thorough follow-up and a multidisciplinary approach in optimizing patient recovery.

## Acknowledgments

This work has been reported in line with the CARE criteria.^[15]^

## Author contributions

**Conceptualization:** Osman Zada.

**Data curation:** Suliman Syed.

**Investigation:** Muhammad Suhaib Hanif.

**Supervision:** Ayesha Haq.

**Writing – original draft:** Osman Zada, Tirath Patel, Suliman Syed.

**Writing – review & editing:** Osman Zada, Tirath Patel, Nikhilesh Anand.
